# Editorial: Technologies in smallholder poultry development: characterization, utilization, conservation, improvement, waste management, and disease control volume II

**DOI:** 10.3389/fgene.2024.1465224

**Published:** 2024-08-01

**Authors:** Abdulmojeed Yakubu, Oladeji Bamidele, Aranganoor Kannan Thiruvenkadan, Moses Okpeku, Senol Çelik

**Affiliations:** ^1^ Centre for Sustainable Agriculture and Rural Development, Department of Animal Science, Faculty of Agriculture, Nasarawa State University, Keffi, Nigeria; ^2^ International Livestock Research Institute (ILRI), Ibadan, Nigeria; ^3^ Department of Animal Genetics and Breeding, Veterinary College and Research Institute, Salem, Tamil Nadu, India; ^4^ Discipline of Genetics, School of Life Sciences, University of Kwa-Zulu Natal, Durban, South Africa; ^5^ Department of Animal Science, Faculty of Agriculture, Bingöl University, Bingöl, Türkiye

**Keywords:** genetics, smallholder poultry, technological advancement, improved productivity, increased food production

Smallholder poultry production systems (SPPS) are very important, especially for low-input farming systems (Bamidele et al., 2023). The SPPS contribute substantially to income generation, job creation, empowerment of women and youths, and improvement in the nutrition and health of farmers, especially in low-and middle-income countries. Despite these benefits, the output derived from poultry species from smallholdings is very low amidst increasing demand for animal-sourced protein as well as the rapid global growth of the human population. Therefore, there is a dire need to embrace modern technologies to conserve and improve poultry genetic resources to guarantee maximum production and productivity of the birds (Hu et al., 2022; Birhanu et al., 2023; Perini et al., 2023; Olaniyan et al., 2024; Zhou et al., 2024).

This second volume (Technologies in Smallholder Poultry Development: Characterization, Utilization, Conservation, Improvement, Waste Management, and Disease Control Volume II) built on the outcome of the first volume (Yakubu et al., 2023), focusing on phenotypic and molecular characterization; quantitative and population genetics; genetic/genomic/proteomic evaluation; analyzing genomes to improve disease control in poultry; animal genomics and infectious disease resistance; application of classical phenotyping methods such as biomarkers and machine learning algorithms to health, nutrition, production, and reproduction including interactions between the environment and poultry species; poultry waste management methods as well as agricultural and environmental issues; and climate-smart approaches to poultry genetic improvement and development. The six articles that contributed to this Research Topic are highlighted below.

The limitation of the slow growth rate in native chickens compared to commercial strains necessitated the work of Tantiyasawasdikul et al. who compared and analyzed the relationship between growth, purine content, uric acid, and superoxide dismutase (SOD) in purebred and crossbred Thai native chickens using 300 birds. The authors observed that the 25% Thai native chicken (TN) group had the highest growth traits at six, eight, and 10 weeks of age, with the lowest in the 100%TN group. As the birds increased in age, there was a decrease in purine content and uric acid in breast meat and liver and SOD in blood. Moderate negative to moderate positive (−0.542 to 0.253) correlation coefficients were found between purine content (total purine, adenine, guanine, xanthine, and hypoxanthine) and growth traits (BW, ADG, and BrC). However, low to moderate positive correlations between uric acid and growth traits (0.348–0.760) and SOD and growth traits (0.132–0.516) were obtained. Three principal components (PCs) were extracted that explained the 86.44% and 86.53% of the total variance in breast meat and liver for selecting animals for optimal balance and also properly separated the purine content, uric acid, SOD, and growth traits. The current findings provided a basis for the genetic improvement of Thai native chickens for high-quality meat yield.

In order to develop a statistical tool for turkey breed’s traceability testing based on meat and carcass quality traits, Salgado Pardo et al. carried out a comprehensive meta-analysis with a total of 75 studies on 37 turkey strains and landraces since the late 1960s. Among the 22 meat and carcass traits investigated, cold carcass weight, slaughter weight, muscle fiber diameter, sex-female, carcass/piece weight, meat redness, ashes, pH24, meat lightness, moisture, fat, and water-holding capacity showed explanatory properties in assigning the birds to their appropriate groups. Carcass traits such as slaughter weight and cold carcass weight were positively correlated. Also, pH showed positive correlations with drip loss and pH24, while water-holding capacity was positively associated with cholesterol and negatively associated with collagen. Fat and ash showed a strong correlation, and both were negatively related with moisture. Strong negative correlations were found as well between meat protein and fat and between collagen and cholesterol. The Mahalanobis distance suggested interbreeding and variety proximity of the birds. It was established that turkey genotype traceability testing can be successfully done using a statistical tool.

The study by Yussif et al. investigated the genetic diversity and population structure of indigenous chickens in Uganda, which constitute over 80% of the country’s chicken resources. Using mitochondrial DNA (mtDNA) D-loop sequences from 344 chickens across 12 populations, the researchers identified 28 polymorphic sites resulting in 32 haplotypes. The population exhibited moderate genetic diversity with a haplotype diversity (Hd) of 0.437 and nucleotide diversity (π) of 0.0169. Most genetic variation (98.39%) occurred within populations, suggesting subtle genetic differentiation influenced by population fragmentation, neutral mutation, random genetic drift, and balancing selection. All haplotypes belonged to the haplogroup-E mtDNA phylogeny, with haplotype UGA01 identified as ancestral. Neutrality tests, including Tajima’s D and Fu’s Fs, and mismatch distribution analyses, indicated a recent population expansion among the chicken populations. The findings revealed a single matrilineal ancestry for Ugandan chickens, originating from a lineage that began in the Indian subcontinent and is widespread globally. Despite low overall genetic diversity compared to other livestock, significant within-population genetic differentiation was observed, particularly in Uganda’s central and eastern regions. The study highlights the importance of conserving genetic diversity within Ugandan chickens due to their adaptability to local conditions. It provides essential insights for designing breeding and conservation strategies to preserve and enhance these indigenous chickens’ genetic resources. Breeding programs should focus on selective improvement to enhance production traits while maintaining genetic diversity. Efforts should also address genetic fragmentation, especially in the northern and western regions, by re-establishing gene flow and improving management practices. Overall, this research offers a foundational reference for the sustainable development and conservation of indigenous chickens in Uganda, emphasizing the need to balance adaptability, farmer preferences, and optimal management strategies.

Under the smallholder farming systems, limited information exists as regards phenotypic and molecular characterization of chickens. This prompted the study of Morris et al. who phenotyped and genotyped a total of 2,573 T451A dual-purpose Sasso chickens reared in emulated free-ranging conditions at ILRI, Addis Ababa, Ethiopia. The phenotypic traits were highly variable and were affected by batch number and sex of the chicken. The genotypes comprised 2.9 million SNPs that were used in the genomic analyses. A largely polygenic mode of genetic control of all phenotypic traits was observed, with the identification of 15 distinct markers that are located in regions harboring relevant annotated genes. These markers were found to be highly associated with growth, carcass traits, NDV titres, IgA levels, and chicken survival. In sub-Saharan Africa, it can be inferred that genetic variability of smallholder chickens may be exploited in selective breeding programs to enhance the productivity of chickens.

In avian species, animal reproduction is influenced by certain environmental parameters. On this basis, Díaz Ruiz et al. investigated the climatological and lunar cycle parameters that greatly affect sperm freezability in roosters. Sperm was obtained from 16 Utrerana breed roosters and replicated 27 times. The motility of the sperm was evaluated and classified into four seminal quality groups (Group 1: Good, Group 2: Satisfactory, Group 3: Acceptable but undesirable, and Group 4: Unsatisfactory) based on FAO guidelines. Minimum temperature, the new moon as moon phase, minimum barometric pressure, and rainfall were the most significant variables to classify an ejaculate in each quality group. The quality of the semen was reported to decrease under lower temperature and precipitation, higher pressure, and a new moon phase. Therefore, the avoidance of these environmental conditions for sperm Research Topic and processing is posited.

The work of Miao et al. investigated the integrative analysis of the ovarian metabolome and transcriptome of the Yaoshan chicken and its improved hybrids. Animal samples for this study were Yaoshan chickens, a local breed in Guizhou, China, and merchant chickens (GYR) with improved egg yields following three-line cross improvement hybridization of Yaoshan chickens. To investigate the regulatory mechanisms behind the differences in laying capability, RNA-seq and ultra-performance liquid chromatography-tandem mass spectrometry (UPLC-MS/MS) were employed to describe the transcriptional and metabolic profiles of Yaoshan and GYR chicken ovaries. At the transcriptional level, the results showed that 288 differently expressed genes were upregulated in Yaoshan chickens while 353 differentially expressed genes were upregulated in GYR chickens. GSEA revealed that inhibiting ECM-receptor interactions and the TGF-β signaling pathway led to higher egg production in GYR hens. Furthermore, metabolomic research revealed that the overexpression of thiamine and carnitine improved hen laying performance. Comprehensive transcriptome and metabolome investigations revealed that thiamine and carnitine were adversely connected with ECM-receptor interactions and the TGF-β signaling pathway, which affect the laying performance of Yaoshan and GYR chickens. Overall, this work identifies variations in the transcriptional and metabolic profiles of Yaoshan and GYR chicken ovaries during peak egg production and proposes new concepts for future research into poultry egg production performance and the related economic benefits.
